# Development of pre-syrinx state and syringomyelia following a minor injury: a case report

**DOI:** 10.1186/s13256-020-02568-6

**Published:** 2020-11-18

**Authors:** Andrea Kleindienst, Tobias Engelhorn, Verena Roeckelein, Michael Buchfelder

**Affiliations:** 1 Dept. of Neurosurgery, Friedrich-Alexander University Erlangen-Nürnberg, Schwabachanlage 6, 91054 Erlangen, Germany; 2 Dept. of Neuroradiology, Friedrich-Alexander University Erlangen-Nürnberg, Schwabachanlage 6, 91054 Erlangen, Germany

**Keywords:** Syringomyelia, Trauma, Pre-syrinx, Treatment, Spinal cord injury

## Abstract

**Background:**

A generally accepted rule is that posttraumatic syringomyelia (PTS) results from spinal cord injury (SCI).

**Case presentation:**

Here, we report the development of syringomyelia without SCI in a 54-year-old Caucasian man following a mild motor vehicle accident. The computed tomography on admission excluded an injury of the spine. Because of neck and back pain, magnetic resonance imaging was performed on day 3 post-injury and demonstrated minimal changes from a ligamentous strain at the cervicothoracic transition. Any traumatic affection of the bone, vertebral discs, intraspinal compartment, or spinal cord were excluded. Some limb weakness and neurogenic bladder dysfunction started manifesting within the following weeks. Repeated MRIs following the accident demonstrated arachnoid adhesions at the C1–2 level and spinal cord edema equivalent to a pre-syrinx state at 12 months and syrinx formation at 24 months. Because of further deterioration, decompression was performed at 36 months.

**Conclusions:**

We conclude that even after a minor trauma PTS can occur and that medullary edema (pre-syrinx state) may precede syrinx formation.

## Background

In the era of implementation of magnetic resonance imaging (MRI) for the diagnosis and follow-up of back pain and spinal cord injury (SCI), medullary abnormalities with a hyperintense T2 signal are increasingly detected. The attribution of these abnormalities as a prominent central canal, hydro- and syringomyelia has been classified by Milhorat [1]. Posttraumatic syringomyelia (PTS) presents with delayed progressive myelopathy often corresponding to spinal segments distant from the level of the original lesion. Experimental evidence confirms that in SCI, local ischemia, hematoma liquefaction, and/or autolytic processes trigger the development of arachnoid scars and PTS [2, 3]. Furthermore, posttraumatic kyphosis and spinal canal stenosis promote the progression of PTS [4, 5]. Mathematical modeling indicates that a phase difference between the arterial pressure pulse in the spinal subarachnoid and perivascular spaces, due to a pathologically disturbed cerebrospinal fluid (CSF) or blood flow, results in a net influx of CSF into the spinal cord [6]. As soon as the intrinsic fluid storage capacity of the spinal cord is overloaded, medullary edema may develop, presenting as a hyperintense T2 signal and referred to as the “pre-syrinx” state [7]. But neither a “pre-syrinx” nor the formation of PTS without any SCI has been reported prospectively. Here, we present the development of a pre-syrinx state at 12 months after a minor trauma without any clinical or radiological evidence of SCI as well as the subsequent syrinx formation at 24 months.

## Case presentation

A 59-year-old Caucasian male patient was referred to us for our expert opinion on the association of a car accident 5 years earlier and the development of syringomyelia. Neither the primary care nor the neurosurgical procedure was performed in our department.

**Fig. 1:**
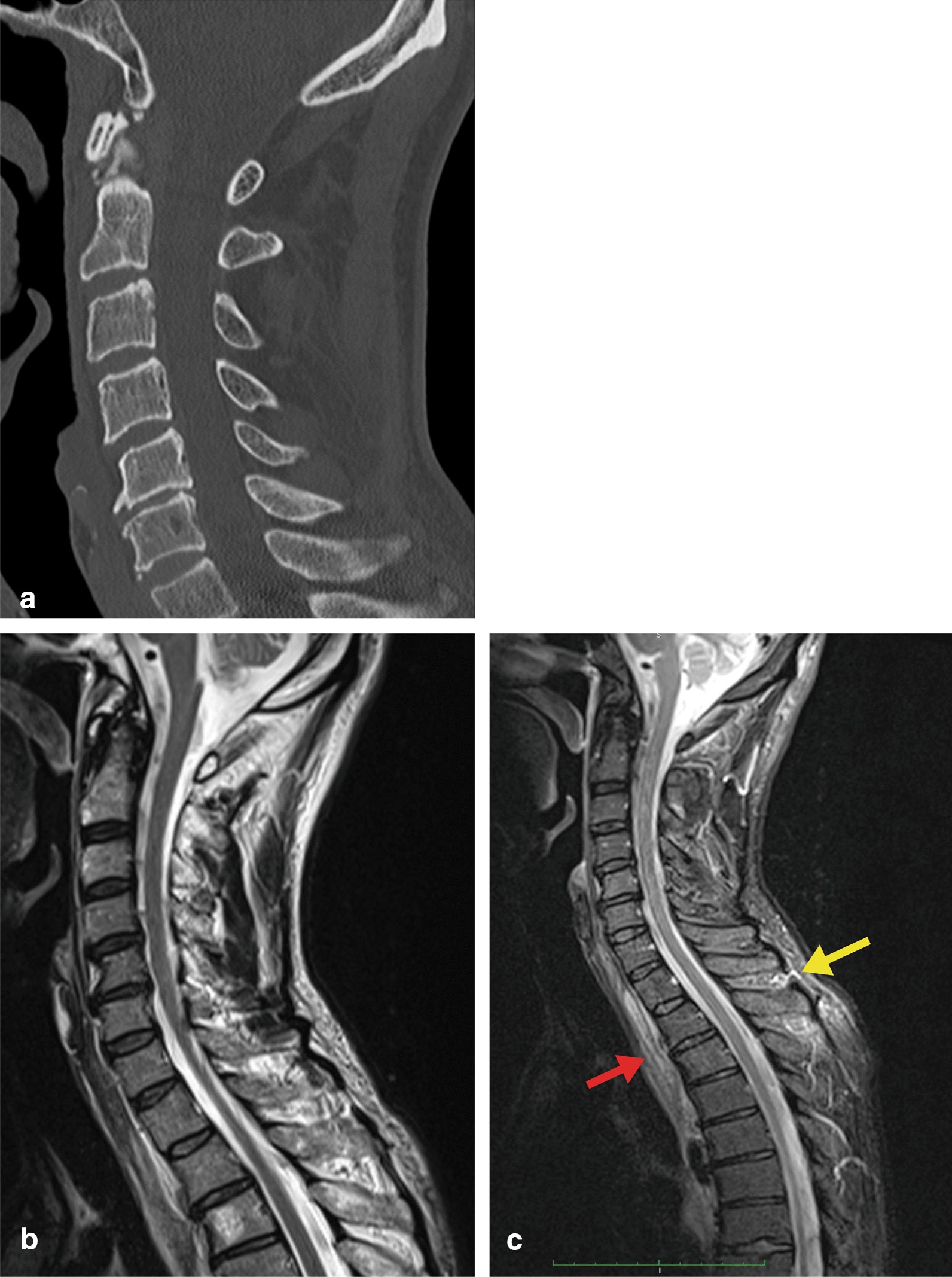
**a** Computed tomography of the cervical spine on admission demonstrates degenerative changes at C5/6. **b** T2-weighted magnetic resonance imaging (1.5 T, 3 mm slice thickness) 3 days later was performed because of neck pain without radicular or medullary symptoms. Degenerative changes were confirmed at C4–7, with mild canal stenosis at C4/5 (10.9 mm) and C5/6 (10.2 mm). A hemangioma at Th3 was observed. Myelomalacia was ruled out. **c** Short-T1 inversion recovery (STIR) magnetic resonance imaging with a large field of view demonstrates subtle prevertebral hemorrhagic effusion within the soft tissue (red arrow) as well as edema between the processi spinosi (yellow arrow) at the cervicothoracic transition

The patient was the driver of a car involved in a collision with a truck. The emergency care was provided by a level II trauma center where he complained of pain in the neck, back, chest, and a right-hand finger. A bruise on the right forehead was surgically dressed. No neurological deficits were observed. The initial polytrauma computed tomography (CT) demonstrated a minor intracranial traumatic subarachnoid hemorrhage. The cranial control CT after 8 hours was normal. The CT of the cervical spine (Fig. 1a) demonstrated degenerative changes corresponding to the patient’s age but no trauma sequelae.

Because of persistent back pain, an MRI of the spine was performed on day 3 post-injury (Fig. 1b) and evaluated as demonstrating degenerative changes and mild canal stenosis at C4/5 (10.9 mm). An incidental hemangioma was found at the level of Th3. *Ex post*, the short-T1 inversion recovery (STIR; Fig. 1c) MRI revealed subtle prevertebral hemorrhagic effusion within the prevertebral soft tissue from C6 to Th 5 (red arrow) as well as edema between the processi spinose (yellow arrow).

The patient could be mobilized only with the support of physical therapists. A global weakness was attributed to pneumonia since the chest x-ray demonstrated a left basal pleural effusion. In the further course, an impaired bladder function was treated as a symptom of a urinary infection. The patient was discharged after 17 days. MRI was not repeated, and an electrophysiological examination was not added.

The patient was re-admitted 2 weeks later because of general weakness, and fever. The neurological examination was normal besides a neurogenic bladder dysfunction lacking any explanation. He was discharged after 2 weeks. A few days later, he reported non-specific sensory alterations. No signs of myelopathy or nerve root compression were found. He was admitted to a rehabilitation clinic. At discharge, hyperreflexia of the right upper extremity and clonus of the left foot were evident.

**Fig. 2:**
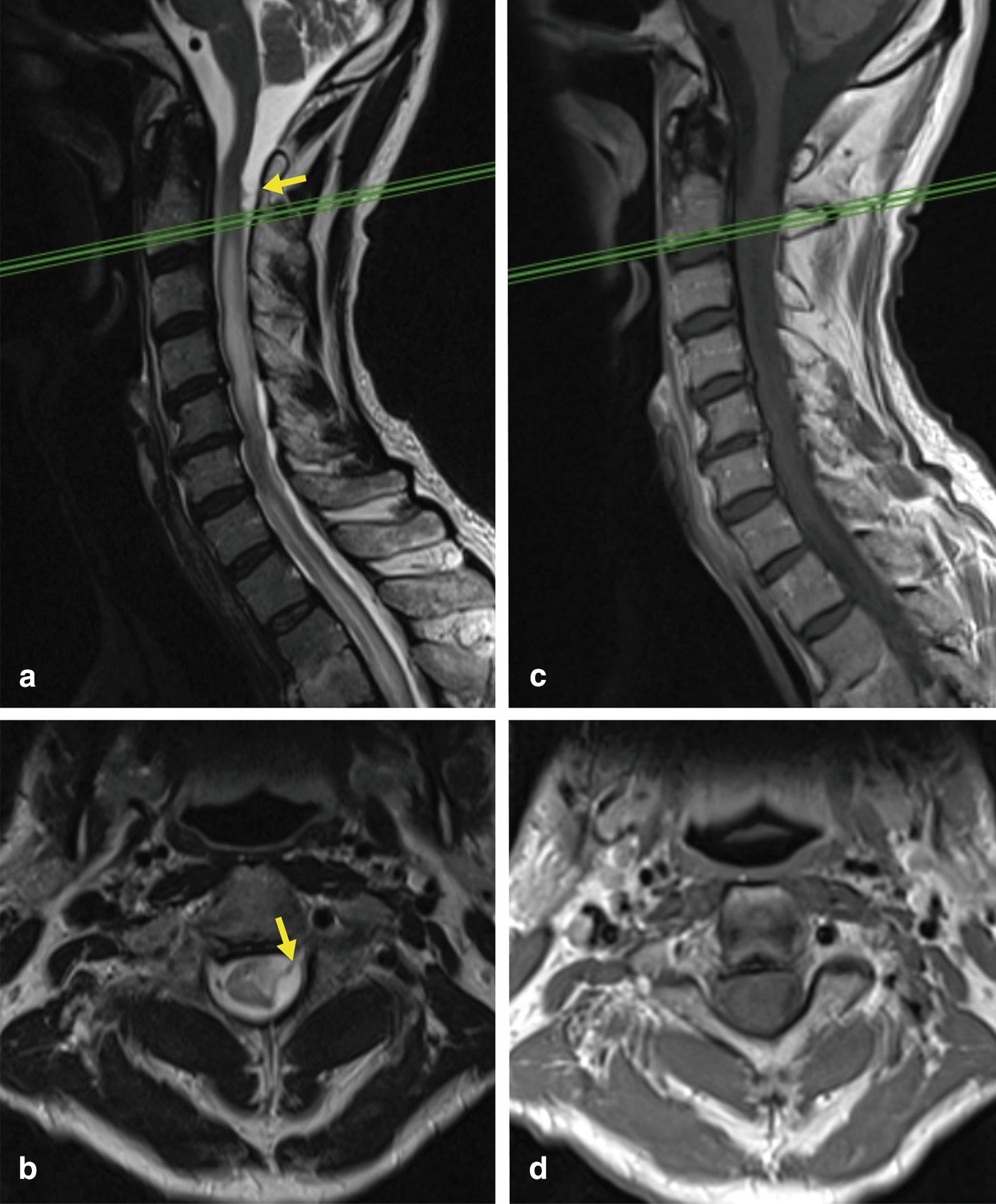
**a**, **b** T2-weighted magnetic resonance imaging (1.5 T, 3 mm slice thickness) 12 months post-injury demonstrated the development of arachnoid adhesions at C1/2 (yellow arrow) and medullary edema at the cervical and upper thoracic level. As an incidental finding, a Th2/3 fracture occurred at the site of the hemangioma. **c**, **d** Following contrast enhancement, an underlying pathology could be excluded

Besides persisting cervical pain, the patient developed a progressive generalized loss of strength and dysesthesia in the hands within the next 6 months. A neurological examination revealed a mild hemiparesis and hypesthesia on the left side, hyperreflexia in the right upper extremity, clonus, and a positive Babinski sign in the left foot. An MRI at 12 months post-injury demonstrated the development of an arachnoid cyst at the C1–2 level and medullary edema at the cervical and upper thoracic level (Fig. 2a, b). T1-weighted contrast-enhanced sequences excluded any neoplastic processes or an infection (Fig. 2c, d).

**Fig. 3:**
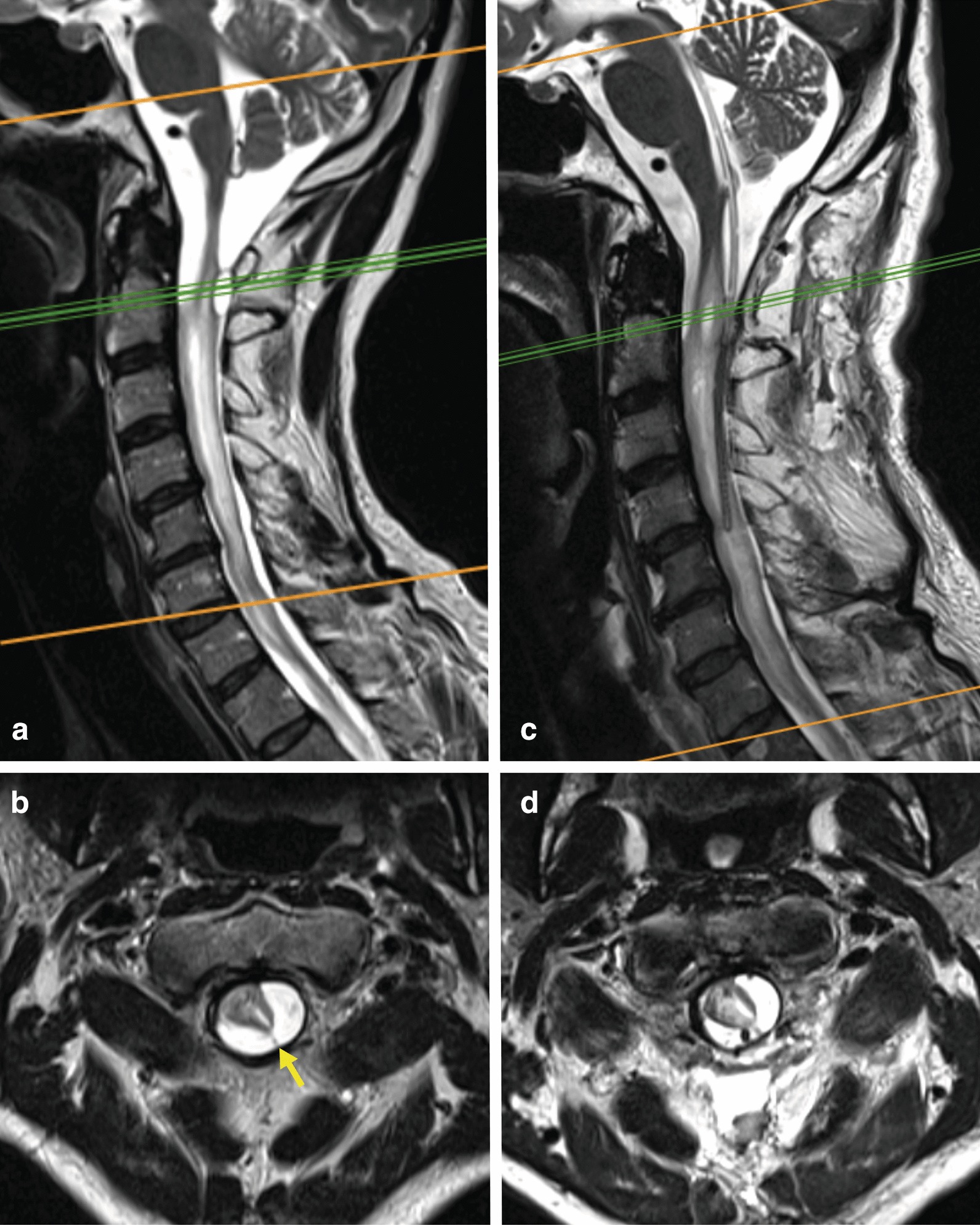
**a**, **b** T2-weighted magnetic resonance imaging (1.5 T, 3 mm slice thickness) 2 years post-injury demonstrated that the arachnoid cyst progressed and that a syrinx cavity developed while the medullary edema was still present. Arachnoid adhesions are marked by the yellow arrow. **c**, **d** The postoperative MRI following decompression, untethering, and drain placement revealed a minimal reduction of the arachnoid cyst but no resolution of the syrinx

**Fig. 4:**
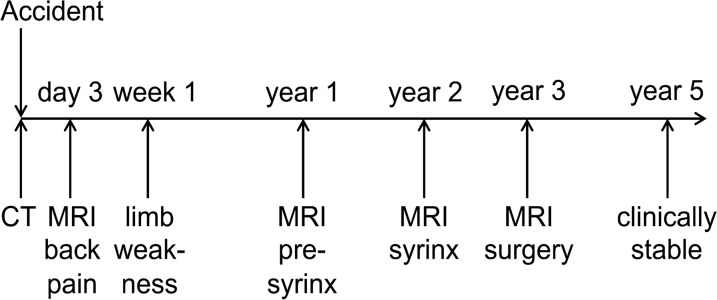
Timeline of events, clinical symptoms, and investigations. *CT* computed tomography, *MRI* magnetic resonance imaging

The further clinical deterioration was accompanied by a worsening of the somatosensory and motor-evoked potentials. The MRI at 24 months post-injury demonstrated arachnoid adhesions at C1/2 and syrinx formation (Fig. 3a, b). Because of electrophysiological deterioration, a decompression at the craniocervical level with a duraplasty was performed at 36 months post-injury. A fourth ventriculo-subarachnoid drain was placed (Fig. 3c, d) and removed a few days later because of a trochlear paresis. Neurological symptoms comprised an accentuated paresis of both arms and the left leg postoperatively. The clinical course was stabilized following continuous rehabilitative training, and the neurological deficits returned to the preoperative status. The patient refused any further surgery. A timeline of the case presentation is shown in Fig. 4.

## Discussion

We present the case of a healthy male patient in his early 50s who was involved a car accident and developed minor symptoms over the course of several days to weeks not acknowledged as being of medullary origin. The CT and MRI after the injury excluding an SCI resulted in a prolonged period of clinical deterioration, the development of a pre-syrinx state at 12 months, and subsequent syrinx formation at 24 months. Below, we discuss the radiological, clinical, and therapeutic evidence and options in detail.

### Radiological findings

The *post hoc* evaluation of the STIR sequence of the MRI 3 days after the injury revealed minimal findings compatible with a ligamentous strain at the cervicothoracic transition (Fig. 1b, c). Since the 1980s, MRI has become the gold standard for assessing SCI radiologically [8] and predicting the risk of SCI even after a mild injury [9]. Discrete traumatic microstructural lesions of the spinal cord can be assessed with diffusion tensor imaging (DTI), T2- and T2*-weighted and STIR sequences within the first 48 hours after the injury although the clinical adoption remains elusive because of complex acquisitions, cumbersome analysis, limited reliability, and wide ranges of normal values [10]. However, in both whiplash injuries resulting from an acceleration-deceleration injury to the spine and “spinal cord injury without radiological abnormalities” (SCIWORA) [11, 12], MRI is outranked by clinical judgment based on the available history [13]. In the presented patient, the ligamentous strain to the cervicothoracic transition was overlooked in the first instance. Nevertheless, given the absence of features distinctive for spinal instability, any therapeutic interventions would have been waived anyway.

In syringomyelia, the particular value of MRI lies in the visualization of arachnoid adhesions (Figs. 2a, b, 3a, b, yellow arrows) or communication of the syrinx with the IV ventricle or the subarachnoid space. Furthermore, medullary edema (Fig. 2a) interpreted as a “pre-syrinx“ can be demonstrated [7].

### Degree of spinal injury and development of syringomyelia

Syringomyelia is increasingly recognized as a factor of neurological deterioration following SCI since MRI has improved the diagnosis. Whenever syringomyelia is diagnosed and the patient's history reveals any type of spinal injury, the question arises as to whether the association is incidental or causative. The correlation between the severity of SCI and the incidence of PTS or the time interval to its onset is controversial [14–17]. Following a standardized experimental impact injury at the level of Th 9/10, 27% of rats develop a PTS within 6 weeks [18]. Symptomatic PTS may occur in less than 10% of patients with SCI, while the prevalence of an asymptomatic syrinx in patients who have been followed for 30 years is approximately 28% [19, 20].

In the presented patient, symptoms compatible with a medullary affection occurred early post-injury but were inconclusive and not followed by an electrophysiological verification or re-assessed by an MRI. Hence, 12 months passed post-injury until another MRI was performed demonstrating medullary edema (Fig. 2a, b) suggestive of a pre-syrinx state potentially preceding a syrinx formation [7]. Experimental evidence indicates that mechanical perturbations of the arachnoid form the basis of syrinx development [6]. However, arachnoiditis may develop in various situations, even in spinal canal stenosis. As a predisposing factor, the patient presented degenerative changes in the cervical spine with a subsequent relative cervical canal stenosis at C5/6 (Fig. 1a).

### Therapeutic options

The decision as to if and when to offer surgery to patients with PTS is based on several considerations and has changed over the past decades [21, 22]. During the 1980s, syringostomy [23, 24] was the preferred treatment option. Thereafter, drain placement was favored [24–33] especially if the syrinx cavity did not collapse intraoperatively [34-36]. Drain complications led to the preference for reconstruction of the subarachnoid space [37], and subsequently decompression and arachnolysis were advocated [35, 38].

In the presented patient, the treating neurosurgeon chose the fourth ventricle as the destination of the drain as suggested by Milhorat *et al.* [39]. Unfortunately, the patient suffered from a trochlear paresis, and the drain was removed 4 days later. The postoperative deterioration of the motor function was stabilized, and the patient recovered to the preoperative status undergoing thorough rehabilitation. Five years later, he was in a clinically stable condition. He was able to walk and climb stairs while suffering from some gait instability due to an impairment of the deep sensory system. Whether the arachnoid lysis prevented any further deterioration and stabilized the neurological worsening remains debatable. From a pathophysiological point of view, surgery may even be reasonable in the pre-syrinx state [7, 14, 40]. However, other authors still favor an exclusively conservative treatment in the event of progressive neurological deterioration [41].

## Conclusion

We prospectively report the development of PTS following an accident initially with no clinical signs of an SCI and a normal MRI of the spinal cord. To the best of our knowledge, this is the first case in the literature to demonstrate the clinical and radiological transition of a normal spinal cord after a minor trauma without any evidence of SCI to medullary edema of a “pre-syrinx” state and further into syringomyelia. The depiction of ligamentous strain at the cervicothoracic transition in the STIR sequences emphasizes the acknowledgment of even subtle MRI findings following any relevant trauma. All therapeutic decisions should be made on an individual case basis given the downhill course of the patient and the evolution of arachnoid scars at the craniocervical junction. Even in the absence of clear evidence of mechanical compromise based on the MRI, one can justify exploring the area at a later stage with the intent of restoring the CSF flow, liberating possible adhesions, and draining existing cavities.

## Data Availability

Data sharing not applicable to this article as no datasets were generated or analysed.

## Abbreviations

PTS: Posttraumatic syringomyelia
SCI: Spinal cord injury
MRI: Magnetic resonance imaging
CT: Computed tomography
CSF: Cerebrospinal fluid
